# Concurrence of bird-related hypersensitivity pneumonitis and systemic sclerosis-associated interstitial lung disease

**DOI:** 10.1093/rap/rkaf037

**Published:** 2025-03-26

**Authors:** Mai Miyahara, Kazuki M Matsuda, Hirohito Kotani, Tomomi Miyake, Asako Yoshizaki-Ogawa, Ayumi Yoshizaki, Shinichi Sato

**Affiliations:** Department of Dermatology, The University of Tokyo Graduate School of Medicine, Tokyo, Japan; Department of Dermatology, The University of Tokyo Graduate School of Medicine, Tokyo, Japan; Department of Dermatology, The University of Tokyo Graduate School of Medicine, Tokyo, Japan; Department of Dermatology, The University of Tokyo Graduate School of Medicine, Tokyo, Japan; Department of Dermatology, The University of Tokyo Graduate School of Medicine, Tokyo, Japan; Department of Dermatology, The University of Tokyo Graduate School of Medicine, Tokyo, Japan; Department of Dermatology, The University of Tokyo Graduate School of Medicine, Tokyo, Japan

Key message

- Hypersensitivity pneumonitis may co-exist with systemic autoimmune rheumatic diseases-associated interstitial lung disease, so a thorough exposure (inhaled and oral) history should always be considered, especially if lung function deteriorates unexpectedly.

Dear Editor, Hypersensitivity pneumonitis (HP) is an inflammatory syndrome affecting the lungs, resulting from repeated inhalation of a variety of antigens, as well as exposure to drugs such as antibiotics, anticancer drugs and immunosuppressants, including methotrexate [1, 2]. HP can present acutely, subacutely or chronically [3] and is classified into nonfibrotic and fibrotic phenotypes [2]. Diagnosing HP can be challenging, especially in the presence of overlapping conditions such as SSc-associated interstitial lung disease (ILD). This case report highlights the diagnostic process and management of bird-related HP in a patient with SSc-ILD.

A 73-year-old female with a 26-year history of limited cutaneous SSc (lcSSc) positive for anti-topoisomerase I antibody (ATA) on 5 mg/day of prednisolone (PSL) complained of worsening of dry cough lasting for 6 months with breathlessness. She was admitted urgently with a fever of 39°C and respiratory failure, with a percutaneous oxygen saturation of 83% on room air. High-resolution chest CT (HRCT) revealed numerous panlobular ground-glass opacities with intralobular reticular opacities (crazy-paving appearance) throughout the lungs, in addition to pre-existing mild subpleural reticular shadows at the bilateral lung bases, consistent with nonspecific interstitial pneumonia (NSIP) observed on HRCT performed 19 months earlier (Fig. 1A). There were no signs of active SSc, such as progression of skin sclerosis, peripheral circulatory disturbances, pulmonary hypertension or renal crisis. Additionally, the patient exhibited unresponsiveness to antimicrobial therapy (ceftriaxone 2 g/day for 3 days). At the time of presentation, her family history was unremarkable, and she had no known allergies. She had never been exposed to methotrexate.

**Figure 1.**
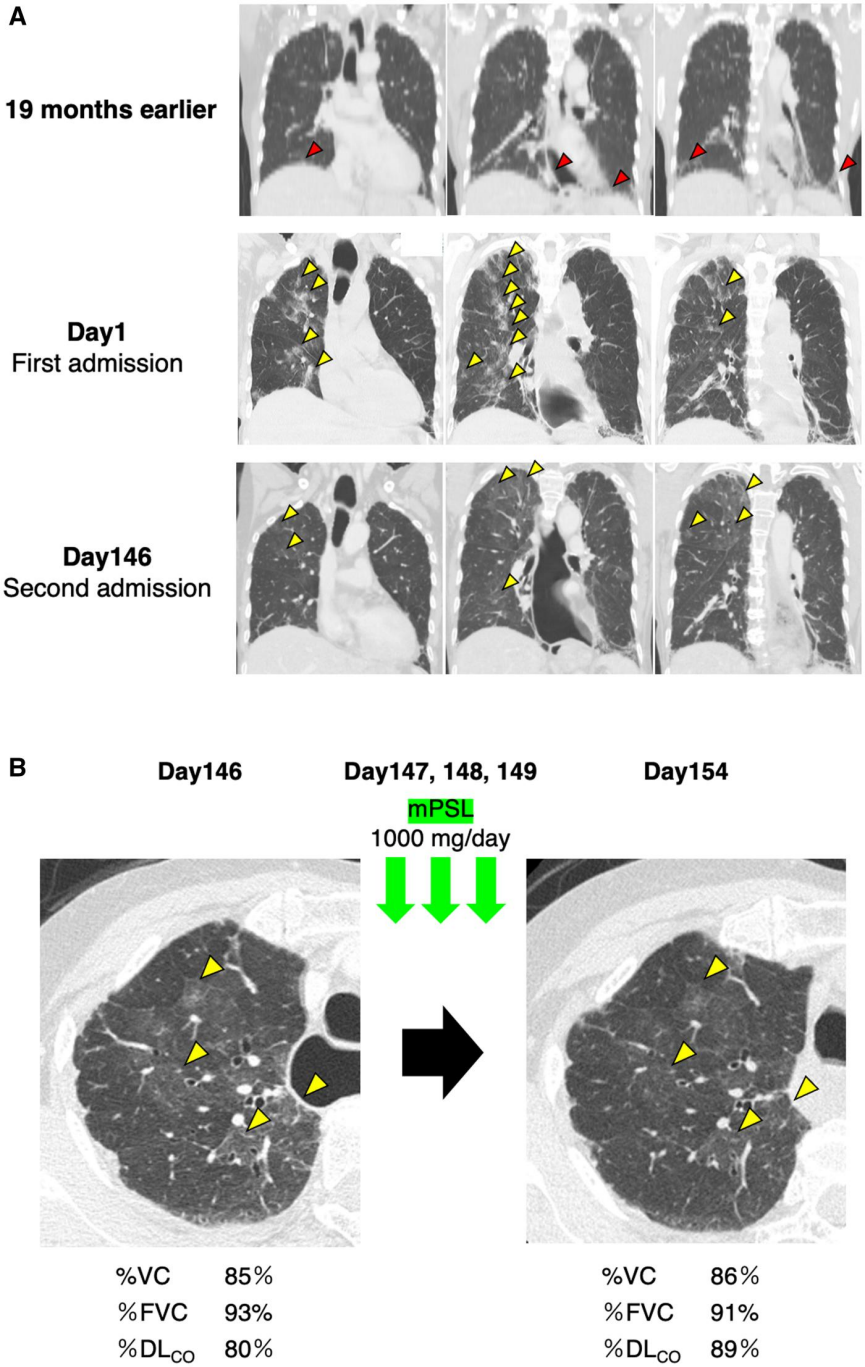
Clinical course of the presented case. (A) Coronal high-resolution computed tomography (HRCT) images at 19 months earlier, and on first and second admission. The time of the first admission was defined as day 1. There were mild subpleural reticular shadows at the bilateral lung bases, consistent with nonspecific interstitial pneumonia, on the baseline (arrowheads in the first raw). On admission, there were numerous panlobular ground-glass opacities with intralobular reticular opacities (crazy-paving appearance) throughout the lungs (arrowheads in the second and the third raws). (B) Comparison of horizontal HRCT images and lung function before and after mPSL pulse therapy during the second admission. Panlobular ground-glass opacities with intralobular reticular opacities improved following mPSL pulse therapy (arrowheads)

Initial treatment with methylprednisolone (mPSL) pulse therapy (1000 mg/day for 3 consecutive days) and subsequent maintenance on 40 mg/day of PSL were provided under the assumption of SSc-ILD exacerbation (Fig. 1A). Her fever and respiratory condition improved with mPSL pulse therapy, and the newly developed panlobular ground-glass opacities with a crazy-paving appearance exhibited temporary improvement. However, her symptoms recurred with tapering doses of oral PSL, leading to her second admission. On admission, she was on PSL 20 mg/day and MMF 1000 mg/day. MMF was introduced in response to her worsening respiratory symptoms, which was presumed to be due to SSc-ILD. Physical examination showed stable vital signs, dry cough and bilateral fine crackles over the lower lung fields. Cutaneous examination revealed no worsening of skin sclerosis nor digital ulcers. Chest HRCT revealed subpleural reticular shadows at both lung bases typically seen in SSc-ILD, showing no progression from previous imaging. However, reticulation and centrilobular nodules were also observed throughout the lung fields, suggesting a different underlying aetiology (Fig. 1A). A thorough review for SSc-related and myositis-related autoantibodies utilizing A-Cube [4], a multiplex autoantibody array assay, revealed no additional findings.

Given the patient’s continuous working in the cleaning industry for many years, which involved exposure to pigeon feathers and droppings, as well as the use of feather bedding, bird-related HP was suspected. Serum positivity of specific IgG antibodies against pigeon (26 mg_A_/l) and budgerigar antigens (23 mg_A_/l) were demonstrated by ImmunoCAP^®^ assay (ThermoFisher Scientific, Waltham, MA, USA). Repeated mPSL pulse therapy achieved clinical improvement, as well as resolution of upper lung ground-glass opacities, and the percent predicted diffusing capacity of the lungs for carbon monoxide (%DL_CO_) increased from 80% to 89% (Fig. 1B). The patient was discharged on oral PSL 20 mg/day. Despite detailed instructions on antigen avoidance, the patient’s dry cough exacerbated post-discharge, indicating ongoing exposure and recurrent HP. She confessed that she was still working as a cleaner and had not removed the feather bedding. After repeated instructions on antigen avoidance, her symptoms improved 11 months after the last mPSL pulse therapy. Currently, PSL has been tapered off without symptom flare-ups.

Diagnosing HP is challenging in patients with overlapping autoimmune diseases such as SSc, which can independently cause ILD. ATA-positive SSc patients often have severe ILD and diffuse skin sclerosis, leading to poor prognosis [5]. However, the patient’s long survival may reflect the better prognosis associated with ATA-positive lcSSc [6]. In this case, HRCT findings were one of the key clues for suspecting HP overlapping with SSc-ILD (Fig. 1B). While both HP and SSc-ILD exhibit lung fibrosis, HP is characterized by centrilobular reticulations predominantly in the upper lung fields, reflecting its transairway pathology, whereas SSc-ILD primarily affects the lower lung fields.

Recently, serological assays such as ImmunoCAP**^®^** assay have emerged as novel tools in the diagnosis of HP [4]. Although cross-reactivity with other bird species has been reported due to shared avian antigens [7], not all potential HP-associated antigens can currently be tested. Serology is also unavailable for drug-induced HP, such as cases caused by methotrexate. Furthermore, false-negative results are common [7, 8]; thus, a negative test does not exclude HP, while a positive test merely indicates past exposure. Therefore, clinicians must maintain a high index of suspicion, carefully considering the exposure history, clinical features, and radiologic findings. Comprehensive assessment incorporating all these elements remains essential for accurate diagnosis [1, 2].

## Data Availability

The data supporting the findings of this study are available from the corresponding author upon reasonable request.
